# Development of an Instrument for Evaluating the Implementation of Occupational Health Programmes in Hospitals

**DOI:** 10.12688/f1000research.165842.1

**Published:** 2025-09-29

**Authors:** L. Meily Kurniawidjaja, Indri Hapsari Susilowati, Yin Cheng Lim, Stevan Deby Anbiya Muhamad Sunarno, Ika Nopa, Abdul Kadir, Helen Andriani, Fachmia Azzahra Aulia Kurnia, Robiana Modjo

**Affiliations:** 1 Department of Occupational Health and Safety, Faculty of Public Health, Universitas Indonesia, Depok, West Java, 16425, Indonesia; 2 Faculty of Medicine, Universiti Malaya, Kuala Lumpur, Federal Territory of Kuala Lumpur, 50603, Malaysia; 3 Faculty of Medicine, Universitas Muhammadiyah Sumatera Utara, Medan, North Sumatra, 20217, Indonesia; 4 Department of Health Administration and Policy, Faculty of Public Health, Universitas Indonesia, Depok, West Java, 16425, Indonesia

**Keywords:** occupational health, hospital, health risk assessment, occupational health services

## Abstract

Hospitals pose a high level of hazard and risks, particularly ones related to occupational health, whose overarching objective is to ensure both healthy workers and a healthy workplace, which can be achieved through effective risk management. This research aims to develop an assessment instrument tailored to hospital occupational safety and health units and their stakeholders across Indonesia to assess hospitals’ efforts to protect workers, with a focus on occupational health services programmes based on identified hazards. It is a qualitative exploratory study. Data collection was made through an in-depth literature review to identify occupational health risk factors in hospitals, focusing primarily on studies involving health risk assessment and/or reports of occupational disease or suspected cases of diseases. This was supported by in-depth interviews and field observations in three hospitals: a government hospital, a private hospital and a teaching hospital. The identified risk factors contributing to hospital workers’ mortality and morbidity were then analysed and formed into measurable indicators. These indicators serve as the basis for developing an instrument prototype to semi quantitatively assess the implementation of occupational health services in the hospitals. The prototype was developed by adapting and modifying the Hospital Safety and Health Management System Self-Assessment Questionnaire from OSHA and then subjected to validation through a 2-day FGD involving experts, practitioners, professional organisations, and other authorities in similar fields of study. The instrument consists of seven main sections; 53 statements with a maximum total score of 159; in addition to guidance. In conclusion, the instrument is a strategic tool that can be used by hospitals to improve the effectiveness of occupational health service implementation in a sustainable manner. Regulatory support, management commitment, and involvement of all elements in a hospital are the keys to realising a healthy, safe, productive workplace for the whole hospital community.

**List of initialisms**
AKHLAK: *Amanah, Kompeten, Harmonis, Loyal, Adaptif, dan Kolaboratif* (Core values for SOEs in Indonesia)
BPJS-TK
: Badan Penyelenggara Jaminan Sosial Ketenagakerjaan
CTS: Carpal Tunnel Syndrome
DT: Diagnosis and Prompt Treatment
DL: Disability Limitation
EAP: Employee Assistance Program
E-medical records: Electronic Medical Records
FGD: Focus Group Discussion
HNP: Hernia Nukleus Pulposus
HRA: Health Risk Assessment
HIV/AIDS: Human Immunodeficiency Virus/Acquired Immunodeficiency Syndrome
HEPA: High-Efficiency Particulate Air
HP: Health Promotion
ICU: Intensive Care Unit
ILO: International Labour Organization
IPC: Infection Prevention and Control
LBP: Low Back Pain
MCU: Medical Check-Up
MRI: Magnetic Resonance Imaging
MSDs: Musculoskeletal Disorders
NCD: Non-Communicable Disease
NICU: Neonatal Intensive Care Unit
OHS: Occupational Health Services
OSHA: Occupational Safety and Health Administration
OD: Occupational Disease
PPE: Personal Protective Equipment
PPI: *Pencegahan dan Pengendalian Infeksi* (Infection Prevention Programs)
PPRA: *Program Pengendalian Resistensi Antimikroba* (Antimicrobial Resistance Control Program)
PICU: Pediatric Intensive Care Unit
REBA: Rapid Entire Body Assessment
RULA: Rapid Upper Limb Assessment
RW: Rehabilitation and Return-to-Work
SOP: Standard Operating Procedure
SP: Specific Protection
SEG: Similar Exposure Group
SOEs: Stated-owned Enterprises
TLDs: Thermoluminescent Dosimeters
WHO: World Health Organization

## Introduction

Hospitals play a vital role in the national health system, with comprehensive service delivery as their core business objective. To support this goal, various resources are needed, especially human resources—both health and non-health workers—who must not only be competent, but also healthy and fit to ensure productivity. However, as workplaces, hospitals pose numerous types of hazards and high levels of occupational health risks. The COVID-19 pandemic (2020–2022) revealed the vulnerability of healthcare workers, many of whom died while performing their duties. Research recorded 1,545 healthcare worker deaths during the first 18 months of the pandemic in Indonesia. ^1^ Therefore, protecting hospital workers through the implementation of occupational health services is imperative. This aligns with the Alma-Ata Declaration and is supported in Indonesia by Government Regulation No. 88 of 2019 on Occupational Health, Article 70, which states that occupational health efforts aim to protect workers so they can live healthy lives free from harmful work-related impacts.

The goal of occupational health is to achieve healthy workers and a healthy workplace. This is achieved through risk management aimed at preventing workers from experiencing health problems or premature death, thereby ensuring productivity and supporting the sustainable growth of businesses or activities. In Indonesia, recorded cases of occupational accidents far outnumber those of occupational diseases (ODs). According to the Ministry of Manpower Republic of Indonesia, the number of reported ODs was 48 in 2019, 81 in 2020, and only 6 in 2021, ^2^ followed by 94 cases in 2023. ^3^ In contrast, BPJS-Tenaga Kerja reported the number of occupational accident cases as 182,835 in 2019, 221,740 in 2021, 234,370 in 2021, and 297,725 in 2022. Furthermore, Ministry of Manpower reported 370,747 cases in 2023 and 462,241 in 2024. ^3, 4^ Such rises are significant.

The number of recorded occupational accident cases in Indonesia is overwhelmingly higher than those of occupational disease, contrary to global data, which show that deaths from occupational diseases are higher than those from accidents. According to ILO and WHO estimates in 2016, of the 1.8 million work-related deaths globally, only 19% were due to injuries, while 81% resulted from work-related diseases. ^5^

This situation is not conducive to worker protection, as unrecognised health risk factors continue to expose workers to health problems ranging from mild to severe, or even death. Therefore, risk management is essential, beginning with the identification of health hazards and followed by risk assessment in the workplace. Health Risk Assessment (HRA) serves as a foundation for occupational disease diagnosis and programme needs analysis, enabling the implementation of health risk control measures as part of corporate accountability in addressing workers’ social and health issues. ^6^

Hence, it is necessary to assess how occupational health is implemented in hospitals. An instrument is needed to evaluate the extent of such protection for hospital workers, with a focus on the implementation of occupational health services based on identified health hazards or risk factors. This research is part of a broader research tree under the Department of Occupational Health and Safety (Departemen Keselamatan dan Kesehatan Kerja), Faculty of Public Health, Universitas Indonesia. It is expected to produce a comprehensive profile of occupational health implementation in Indonesia and to provide recommendations to inform national policy development.

## Methods

This is a qualitative exploratory study. Data collection was conducted through an in-depth literature review to identify occupational health risk factors in hospitals, primarily drawn from studies involving health risk assessment (HRA) and/or reports of occupational disease cases or suspected cases. This was followed by in-depth interviews and field observations. The identified risk factors contributing to hospital workers’ mortality and morbidity were then analysed, and the results used as the basis for developing a semi-quantitative measurement instrument (scoring system) prototype to assess the implementation of an occupational health service programme in hospitals. The prototype was developed by adapting and modifying the Hospital Safety and Health Management System Self-Assessment Questionnaire from OSHA. The prototype was then subjected to validation through a 2-day FGD involving experts, practitioners, professional organisations, and other authorities in similar fields of study.

The study was conducted from May 2024 to March 2025. The focus was on accredited Type B general hospitals, based on the consideration that Type A hospitals are too large in scale, while Type B ones generally meet adequate standards in terms of resources and management compared to Type C ones. Three Type B hospitals were selected, chosen based on accessibility and in order to represent government, private, and teaching hospitals.

The following three research flow steps were taken to develop the prototype.

### Step 1

In the instrument development process, qualitative interviews were conducted with informants comprising the heads of the Hospital Occupational Safety and Health Unit (Hospital OSH Unit); Infection Prevention and Control (IPC); general medical service managers (covering basic medical services, dental and oral health, and maternal and child health/family planning); emergency department managers, medical support service managers; nursing managers; and, where applicable, quality committee chairpersons. The number of informants was determined based on data saturation. The inclusion criteria were that they had worked in their current position for at least one year and had signed an informed consent form.

### Step 2

The qualitative data collected were analysed using a thematic analysis approach. This method was chosen for its ability to explore and understand the various risk factors emerging from the qualitative data, enabling the identification and analysis of key aspects related to the research problem, namely five groups of health risk factors in the workplace (related to the environment, ergonomics, workers’ somatic conditions, worker behaviour, and work organisation and culture). Thematic analysis allowed for systematic organisation and interpretation of the data, facilitating in-depth insights from the respondents studied.

The identified risk factors contributing to hospital workers’ mortality and morbidity were then analysed, with the results used as the basis for developing a semi-quantitative measurement instrument (scoring system) prototype to assess the implementation of occupational health services programmes in the hospitals.

### Step 3

The prototype was then subjected to validation through a 2-day FGD. The FGD participants included academicians, experts, practitioners, representatives from professional organisations, hospital directors and their teams, the Ministry of Health and Ministry of Manpower as the regulators. The number of participants was adjusted according to data needs and saturation. Inclusion criteria for the FGD participants were based on the principle of appropriateness to the study objectives, with a minimum of one year in their current position and having signed an informed consent form. The final result was an instrument consisting of indicators to assess the implementation of occupational health service programmes (Appendix 1), together with related guidelines (Appendix 2).

### Ethical consideration

This study has gone through a review process and was approved by the Institutional Review Board from teaching hospital Rumah Sakit Universitas Indonesia (approval number: S-091/KETLIT/RSUI/VII/2024). Written informed consent was obtained from all participants, including informants for in-depth interviews and stakeholders in focus group discussions (FGD). The consent process ensured that participants were fully informed about the study’s purpose, procedures, potential risks, and their right to withdraw at any time without consequences. No animal or human biological materials were involved in this study. All data were anonymized, and identifiable information was securely stored to protect participant confidentiality.

## Results and discussion

### Analysis of Health Risk Assessment (HRA) implementation in Type B hospitals

The findings from the in-depth interviews, observations and focus group discussion (FGD) indicated that all the hospitals reported having conducted health risk assessments (HRAs). However, these were most likely to have been limited to hazard identification without progressing to actual risk level evaluation. The hospital results were presented as internal documents, but we were not allowed to have copies of these. The health hazards identified originated from five main sources: (1) the work environment, including significant physical, chemical, and especially biological hazards; (2) ergonomic factors, such as technical risks, organizational issues, task-related demands, individual factors, and psychosocial or psychological stressors; (3) the somatic conditions of workers, particularly their health status; (4) health-related worker behaviours, including lifestyle and work patterns; and (5) work organisation and/or workplace culture ( Table 1). All these identified health risks have the potential to negatively impact workers’ health. Therefore, it is strongly suggested that the next critical step for hospitals is to assess the risk levels and determine whether the risk factors are being adequately controlled. Risk control measures should be integrated into hospitals’ occupational health service programmes to prevent work-related health problems.

**Table 1.** Identified health hazards in hospitals.

No.	Hazard	Hazard identified	
		In-Depth interviews and observations	FGD
1.	Environmental Hazards		
	Physical Factors	Noise, electrical discharges, cold stress, heat stress, needle and sharp equipment, radiation.	Electrostatic, with many incidents related to grounding, common in cold areas such as cold storage for food and surgery theatres. Cold stress in cold storage. Heat stress, especially in laundries and kitchens. Radiation, including electromagnetic radiation in hospitals that provide MRI. In addition, vibration hazards such as for dentists (hand-arm local vibration).
	Chemical Factors	Alcohol, reagents, anesthetic gases, antiseptics, formalin, desinfectants, chlorine, and used batteries.	Gas exposure from nuclear medicine (for chemotherapy), ethylene oxide for sterilisation, wastewater treatment plants, and generators.
	Biological Factors	Blood (Hepatitis B, C, D, HIV); aerogen transmitted infections (mumps, influenza, rubella, tuberculosis, measles, COVID-19); orally transmitted infection (urine and feces), e-coli, hepatitis A, typhoid, staphylococci, shigella, amoeba, scabies, nosocomial, mouldy ceilings.	The same hazards identified by FGD. Not all hospitals regularly implemented antimicrobial resistance control program (PPRA) committees to conduct culture and resistance tests, infection prevention programs (PPI), and swab tests from the air and walls in the patients' treatment rooms/areas.
2.	Ergonomic Hazards		
	Technical Factors	Awkward positions (risk of HNP, LBP, CTS); extreme cold locations (surgical rooms, food cold storage, specific instrument rooms). Unsuitable ergonomic design (chairs and tables that are not adjustable in administration and radiology rooms, and action rooms that are narrow and in which one is not free to move (ultrasound); patient beds that are not adjustable). Uneven, unstable or slippery surfaces (uneven roads, for example, due to cables that are covered).	Awkward positions are very common in hospitals, not only for health workers, but also in relation to manual handling in nutrition departments (cooking for the hospitals) and laundries (removal of linen), surgical rooms, ICU, NICU, PICU, food cold storage, and in toilets, washing areas and kitchens.
	Organisational Factors	Heavy loads, repetitive work, time pressure, lack of equipment or provision of inadequate equipment (resulting in nurse and patient complaints), poor maintenance of equipment, lack of initial and follow-up training, insufficient workers for the amount of work, poor workflow design, poor information processes, and non-provision of PPE.	In some hospitals, evaluations are made before purchasing equipment and vendors. Some hospitals have only partially implemented guidelines, SOPs and communication networks, and clear SOP flows not always posted in respective workplaces.
	Factors Related to Task	Manual handling of patients (weight or part of the patient's weight), such as lifting, pushing, pulling, transferring, carrying, or other activities. Awkward postures or movements, such as bending, twisting, raising arms, bending wrists, overreaching, or over exertion. Repetitive activities/handling; prolonged standing or sitting, such as in administrative work, combined with screen working.	-
	Individual Factors	Work shifts alternated between employees without reporting this to supervisors.	Fingerprints are not used at all hospitals, so that workers can change shifts without reporting this. Shift changes can occur when colleagues are sick, which could be regulated if there was a limit on overtime, but sometimes it is overlooked.
	Psychological and Psychosocial Factors	High job demands, threat of COVID-19 infection, social rejection, lack of control over workers’ individual work, interpersonal relationships, lack of respect, shortage of assistance, interpersonal conflict, harassment.	Some governmental hospitals already have an AKHLAK culture (abbreviation of SOEs program in Indonesia), and respect in workplace learning modules, especially those under SOEs. Violence or bullying in the workplace is still found, even though it has been regulated by ethics and OSH committees. There remains a culture of reluctance to report, a situation which needs to be improved.
3.	Somatic Hazards		
	Health Status	Allergies, hypertension, dyslipidemia, obesity, prior history of MSDs	There is a pre-placement health examination. Some hospitals already have regular health checks, so it is hoped that somatic risks can be detected early and prompt treatment given. But not for all employees, and rarely health examinations are hazard-based.
4.	Behavioural Hazards		
	Lifestyle and Work Style	Life style: lack of exercise, sedentary work, junk food and fried food eaten, poor sleep quality and quantity. Carelessness, poor habits, lack of responsibility in obeying the SOP. Lack of competence, but not willing to be transparent, so no opportunity to improve this.	Staff tend to consume a lot of unhealthy food and drinks during meetings.
5.	Work Organisation and Work Culture Hazards		
	Work Organisation	Time pressures; work-related loads (shift work, night work, irregular working hours); contradictory work instructions from doctors, senior nurses, nursing service management, or residential area management; work organisation which is not ideal (working-time arrangements), too much responsibility, insufficient aptitude or lack of professional experience, unclear task assignment, incursions into workers’ leisure time; unfavourable environmental influences (noise, electrical discharges, cold, heat, and draughts); complex technical systems exceeding human capacity to think and process information (e-medical records and system), insufficient staff, long distances to cover, or rambling corridors and similar wards, residential areas or floors.	E-medical records are a scourge for senior doctors, with adjustment and time needed to build a difficult culture, despite repeated socialisation and education. There remain problems related to the behaviour of workers, who are reluctant to learn AI and other innovations. E-medical records have relatively few obstacles, and if there are problems with the internet system, there are existing manual SOP services in some hospitals. For long distances, bicycles have generally been facilitated, which help not only with long distances, but also downhill and uphill road conditions. Some workers have to work during rest hours because of the lack of manpower, leading to high workloads. There is no material or non-material appreciation for this extra work outside working hours. Some people bring their stress from home to work. Middle management and above do not receive overtime pay, even if they complete work during breaks or after office hours. There are also individuals who do not have the related competence but have been forced to occupy higher positions.
	Work Culture	Poor career opportunities; lack of recognition, gratification and reward; high physical and mental loads and strains; inadequate personnel leadership; lack of relevant information; lack of support and assistance; difficulties in communication and interaction; social conflicts, harassment, bullying, violence, and discrimination; poor working atmosphere, with constant interruptions and disturbances from colleagues, patients or relatives, and residents; inadequate consideration of compatibility between family and job; lack of support; little or poor communication; conflicts with superiors and colleagues; structural changes in the company; being alone in the workplace (during night and weekend shifts); fear of tasks, blame and sanctions; fear of making mistakes; lack of social and communicative skills; family conflicts.	For workers to continue their education, scholarships and fellowships are provided, although more are given to medical personnel; for example, medical specialist education. There are also work status promotion programmes. There are awards. The best employees proposed by each unit are selected, with assessments and awards for the work units with the best performance. There are psychosocial hazards; most likely from patients, but also from coworkers. For example, dealing with patients and families’ complaints, with some patients not accepting explanations or conditions, and some nurses even beaten. Complaints from patients related to services, facilities and infrastructure, which can reduce ratings on the internet. Consequently, if a rating drops, there will be pressure from the leaders or supervisors, meaning workers in the relevant unit will have to make improvements. There are still cases of verbal bullying. Reported harassment behaviour will be investigated and evaluated. However, the culture of harassment reporting needs to be improved.

### Analysis of occupational health service programme implementation in Type B hospitals

The assessment of occupational health service programme implementation was conducted following the identification of health risk factors. Four key aspects of the programme were examined: (1) documentation of existing programmes; (2) analysis of programme adequacy using the 5-5 Model, a framework that integrates five sources of hazards with five levels of disease prevention, which assesses alignment with the identified risk factors and the needs for occupational disease prevention programmes across five levels of prevention — health promotion (HP), specific protection (SP) against risk factors from five sources, early diagnosis and prompt treatment (DT), disability limitation (DL), and medical rehabilitation including return-to-work (RW) programs; (3) recommended occupational health programmes (see Table 2); and (4) the decision-making process for programme development, assessed through hospital leadership’s commitment as reflected in policy, and worker involvement (see Table 3).

**Table 2.** Assessment of occupational health service programme implementation: Existing versus suggested programmes.

No.	Hazards identified	Existing OH services programme	Existing model 5-5					Suggested OH services programme
			HP	SP	DT	DL	RW	
A	Environmental Hazards							
1	Biological	Training on spill kit usage	V					
		Standard operating procedures (SOP) for patient admission and transport		V				SP: Monitoring of microbial growth in ventilation system ducts and filters; engineering control, with immediate response to findings/reports
		Medical check-up (MCU) and treatment			V	V		DT: Employee health clinic
		Medical rehabilitation and return-to-work program					V	RW: Occupational medicine specialist, physician
2	Physical	Training: education on the use of personal protective equipment (PPE)	V					HP: Hazard communication materials, SOP socialization and mentoring
		Injection SOP; SOP for needlestick injury reporting and management; use of safety needles in specific units; measurement of noise, heat and radiation levels; work hour regulation in high noise areas; provision of PPE (aprons and TLDs); work schedule regulation related to radiation exposure; existence of a risk register with regular evaluation		V				SP: Exposure measurement; SOP for 100% compliance in hazardous procedures and PPE use; engineering control; immediate response to findings/reports
		Medical check-up (MCU) and treatment			V	V		DT: Employee health clinic
		Medical rehabilitation and return-to-work programme					V	RW: Occupational health physician or occupational medicine specialist
3	Chemical	Training	V					HP: Hazard communication materials, SOP socialisation and assistance
		SOP for hazardous materials handling; hazardous material symbols, MSDS, PPE, with a risk register that is regularly evaluated	V	V				SP: Exposure measurement; SOP for 100% coverage of hazardous actions and PPE use; engineering control; immediate response to findings/reports
		Medical check-up (MCU) and treatment			V	V		DT: Employee health clinic
		Medical and occupational rehabilitation					V	RW: Occupational health physician or occupational medicine specialist
B	Ergonomic Hazards	Training						HP: Hazard communication materials; SOP dissemination and mentoring
		SOP for patient lifting and transfer; appropriateness of work equipment		V				SP: Ergonomic assessment (REBA, RULA); SOP for 100% coverage of hazardous actions and PPE use; engineering control; immediate response to findings/reports; regularly updated risk register
		Medical check-up (MCU) and treatment			V	V		DT: Employee health clinic
		Medical and occupational rehabilitation					V	RW: Occupational health physician or occupational medicine specialist
C	Somatic Hazards	Metamorphite training programme	V					HP: Fit to work concept; socialisation and mentoring
		Workplace exercise		V				SP: Adequate exercise frequency and duration; avoidance of sedentary behaviour; application of the 5A principle for all workers (on a rotating basis); immediate response to findings/reports; regularly maintained risk register
		Medical check-up and treatment			V	V		DT: Employee clinic, hazard-based medical check-up (MCU), and fit to work assessment
		Medical and occupational rehabilitation					V	RW: Occupational health physician or occupational medicine specialist
D	Behavioural Hazards	Training						HP: Healthy behaviour education (life & work); socialisation and mentoring involving health promotion officers
		Supervision		V				SP: Target consensus, evaluation and consequences, and risk register
		Medical check-up and treatment			V	V		DT: Employee clinic and hazard-based medical check-up (MCU)
		Medical and occupational rehabilitation					V	RW: Occupational health physician or occupational medicine specialist
E	Work Organisation and Work Culture Hazards	Work stress management training	V					HP: Provide a psychologist or employee assistance program (EAP) specialist
		Measurement of job satisfaction and organisational culture; employee satisfaction survey; regulations on working hour limits and shift scheduling without long shifts		V				SP: Leadership materials for management; fatigue and stress management for workers; immediate response to findings/reports; regularly updated risk register
		Work stress screening, medical check-up (MCU), and treatment			V	V		DT: Work stress questionnaire, involving a psychologist or psychiatrist
		Medical and occupational rehabilitation					V	RW: Occupational health physician or occupational medicine specialist, EAP specialist, psychologist, and employee assistance program (EAP) specialist

**Table 3.** Assessment of decision-making process for occupational health service programmes.

	HRA	Programme	Findings
**Policy**	Risk register policy	OHS programme policy	
**Leadership**		Availability of facilities and infrastructure for monitoring and evaluation processes	Leadership training has been conducted based on employee level through the Employee Orientation programme. However, monitoring has not yet been implemented, while the training is still being conducted. The Jakarta Regional Government provides 40 hours of annual training, which can be run with adaptable topics. Regarding hospital accreditation, there is an incentive for hospitals to implement OHS as part of quality and patient safety measures, including how leadership can drive OHS programmes effectively.
**Worker Participation**	Workers can complete online/form risk register	Workers can propose OHS programmes	All units are involved, with programmes determined by workers through a designated channel via the sub-division head. The worker participation process is effective. Decision-making originates from the bottom-up, not from the Hospital OSH Unit; instead, proposals are submitted to Hospital OSH Unit for further action.
**Contractor Involvement**			Contractors can also be involved. There was a case of a workplace accident triggered by third-party work, highlighting the need for this instrument to ensure that third parties comply with occupational health and safety (OHS) standards.

The data compiled from the hospitals studied indicate that the existing occupational health service (OHS) programmes for controlling identified hazards cover the five levels of disease prevention related to exposure. Following verification and confirmation through the FGD, we conclude that all hospitals should ideally be able to implement similar programmes. However, based on the findings and analysis, certain OHS programmes are recommended and have been arranged sequentially according to the five levels of prevention for effective hazard control (see Table 2).

1. Health Promotion
  a. Enhance workplace health promotion by preparing communication materials, including hazard communication content and SOPs for critical tasks, as well as materials on healthy lifestyles and stress management.
  b. Counselling materials on risk factors should not focus solely on biological hazards, which have been well-managed by the Infection Prevention and Control (IPC) teams, but should also include other risk factors such as chemical hazards, which have not been sufficiently detailed in hazard communication, together with physical risks such as radiation and heat.
  c. Hazard communication tools should include a list of hazards and hazard maps; SEGs (similar exposure groups); and predictive area health mapping. Ideally, current area health maps should also be available. However, the findings show that only one unit has created individual body health mapping, which has not yet been aggregated into the collective data.
  d. To improve workers’ physical and mental health status and their work capacity, management should involve health promotion officers, psychologists or employee assistance program (EAP) specialists, and psychiatrists. It was found that only some units employ psychologists and/or psychiatrists for workers experiencing depression, anxiety, or other forms of distress, which are usually only handled by direct supervisors. Periodic mental health screenings using questionnaires should be provided across all hospitals.
  e. It is necessary to establish clear methods for socialisation, mentoring procedures, and supervision in the implementation of SOPs.
2. Specific Protection
  a. Monitoring of microbial growth in ventilation system ducts and filters should be conducted, in addition to air sampling. This recommendation is based on findings in one hospital, where water stains were observed on the ceiling of a large outpatient waiting area (estimated at over 15 x 10 m ^2^). Despite repeated annual repainting and even the replacement of rotted ceiling panels, the issue, suspected to be related to the piping system, has yet to be fully resolved. Water stains were also found on several walls and corridors. Water seepage, like stagnant water, serves as a breeding ground for microbes.
  b. Measurement of hazard exposure levels, especially physical, chemical and ergonomic factors, needs to be improved. The measurement results can be used to assess risk levels and prioritise control measures. The study found that risk assessments were not conducted comprehensively and were limited to a few units, often performed by unit staff without the support of experts or the hospital’s OHS implementation unit.
  c. It is suggested that ergonomic risk assessment use well-established measurement tools that are relatively simple and considered valid and reliable, such as REBA and RULA. It was found that only one hospital unit had conducted such assessment, in relation to mismatches between workers and work tools, equipment or workstations.
  d. Implementation and supervision of SOPs, including PPE use, need to be strengthened, with a target of 100% compliance to prevent hazardous actions.
  e. Engineering/technical controls, beyond administrative controls such as SOPs, should be enhanced in all relevant aspects, including environmental and ergonomic risk factors. It was found that advanced technical infection control measures have been implemented by two hospitals inspected, such as improved ventilation, HEPA filters, and specialised washing machines. However, technical controls for transferring bariatric patients were only found in one hospital, and the equipment was not functioning effectively because the European-made patient lifting equipment was too large for the Japanese-standard corridors. In addition, non-ergonomic desks and chairs were still found, especially in administrative areas where they were not typically visible, even though adjustable chairs were commonly used in other units.
  f. Workers’ health behaviours need improvement through education on non-communicable diseases (NCDs), the Fit to Work concept, socialisation, and mentoring—ideally involving health promotion officers. The assumption that all hospital staff, both medical and non-medical, are well-versed in healthy lifestyles is not entirely accurate. The interviews and field observations revealed that health behaviour-related risk factors significantly affected workers’ health and capacity, such as obesity, poor sleep quality and duration, working while fatigued, and shift swapping between employees without notifying supervisors, despite the completion of digital attendance.
  g. Specific protection strategies for both lifestyle and work behaviour should be considered, with targets established through consensus, and accompanied by defined evaluation methods and consequences. Behavioural risk factors should also be included in the risk register to ensure they are detected and addressed.
3. Early Diagnosis and Prompt Treatment
  a. Periodic health examinations should be conducted regularly, especially biological exposure assessments, as these represent a primary concern for both healthcare and non-healthcare workers in hospitals. This recommendation is based on our findings that medical check-ups (MCUs) are sometimes only conducted for selected high-risk units, such as ICUs, operating rooms, haemodialysis, inpatient wards, and outpatient clinics, while workers in laundries, cafeterias, and administrative offices are not guaranteed annual check-ups, with some never having been given one. It is widely known that infection can be transmitted through airborne routes in hospitals, in addition to direct contact with patients or sharps, which potentially affect all workers, both clinical and non-clinical.
  b. Establishing an employee clinic within hospitals in the form of a primary healthcare facility is strongly suggested. This proposal also emerged during the FGD and was supported by the health authorities and professional organisations. Comprehensive occupational health efforts, including promotive, preventive, curative and rehabilitative services, are implemented in such facilities. Furthermore, immediate treatment requires an accessible employee clinic within the hospital. However, under the current National Health System, hospitals are categorised as referral healthcare facilities, so workers must first visit a primary healthcare facility outside the hospital, navigate a lengthy process involving registration, wait for consultation, and then collect medication. This process is inefficient in terms of time, energy and cost, and causes discomfort and demotivation among hospital staff. Moreover, employees’ medical history data, regarding both occupational and non-occupational diseases, are poorly recorded within hospitals.
  c. The involvement of an occupational medicine specialist is essential for a more accurate diagnosis of occupational diseases, given the complex risk factors affecting both healthcare and non-healthcare workers in hospitals.
4. Disability Limitation
  a. Disability limitation is well managed by intensive treatment programmes within the hospitals. However, if the required facilities are not available, it is strongly recommended to make use of the referral system to another hospital, making the process more effective and efficient.
  b. Immediate response to findings and reports should be ensured. The in-depth interviews with operational workers (both healthcare and non-healthcare staff ) and the field observations revealed complaints about delayed or even lack of responses to reports, despite the presence of a risk register system. Uncontrolled hazards may materialise and lead to health problems, disability or even death. In the long term, this undermines the development of a safety culture, in addition to causing short-term losses.
5. Rehabilitation
  a. The return to work programme is essential, alongside medical or physical rehabilitation, with the presence of an occupational medicine specialist valuable, particularly for healthcare workers with health limitations due to occupational or non-occupational diseases. They can assist in determining appropriate tasks for such workers, especially in roles affecting patient safety, while also protecting their health from further deterioration; for example, nurses infected with tuberculosis, hepatitis B or HIV/AIDS.

### Analysis of the decision-making process for occupational health services programmes

We continued the research by examining the decision-making process followed by the Hospital OHS Unit. The components assessed were (1) Policy; (2) Leadership; (3) Worker Participation; and (4) Contractor Involvement (see Table 3). Particular attention was paid to leadership commitment as reflected in hospital policies and worker involvement.

We found that written policies are in place. They require all hospital staff to report identified hazards through the risk register, whether from periodic assessments or unplanned findings. Each hospital has also issued written policies mandating the implementation of occupational health services for its personnel. Additionally, hospital accreditation policies in Indonesia have encouraged the implementation of occupational health programmes, including the obligation for leadership to demonstrate commitment in promoting and supporting these. This is the same as the neighbouring country such as Malaysia, whereby it is the responsibility of employers to have the occupational health program for the employees.

Leadership commitment is reflected in the implementation of leadership training aimed at reducing risks associated with work organisation and workplace culture. However, no monitoring or evaluation has been conducted to assess the impact of this training on leadership. In implementing general occupational health programmes such as workplace exercise, leaders are encouraged to participate actively on a rotating basis to promote staff engagement. Additionally, the development of health education materials should be needs-based, feasible, and accommodate workers’ preferences (in line with their needs). Therefore, material development should be led by the unit head, undertaken jointly with worker representatives, and facilitated by the Hospital OSH Unit.

In the process of hazard identification, planning, and determining occupational health service programmes, the Hospital OSH Unit receives reports and suggestions from all hospital workers and contractors, who can report hazards online via the risk register, or offline using a form. They can also propose occupational health service programmes through their respective unit leaders, who then submit these to the Hospital OSH Unit.

### Development of an instrument for evaluating the implementation of occupational health programmes in Type B hospitals

Analysis of the implementation of HRA and of OH Services, together with the process of determining an OH services programme, has produced an instrument for measuring the implementation of such a programme (Appendix I). The format of the instrument was developed by adapting and modifying the Hospital Safety and Health Management System Self-Assessment Questionnaire, with written permission to do so obtained from the authorised OSHA party. As stipulated by OSHA, the scope of this document is recommendation-based and merely contains relevant information. The document is not a standard or regulation, nor is a legal obligation.

The instrument was designed in the form of statements to help Hospital OSH Units assess the implementation of occupational health services. The instrument consisted of 53 statements in seven sections, with a maximum total score of 159. Each question, divided into the seven groups below, was given a score of between 0 and 3.

1. Management Support in the Process of OH Services Implementation (6 statements)
2. Worker Participation and Leadership Involvement (6 statements)
3. Hazard Identification and Health Risk Assessment (12 statements)
4. Health Hazard Control (7 statements)
5. Disease Prevention (8 statements)
6. Education and Training (9 statements)
7. Occupational Health Service Programme and Evaluation (5 statements)

The statements cover the implementation of occupational health in hospitals in an effective occupational health management system.

To make it easier for the respondents to respond to the statements, they were asked to review the “Guidance for Completion” column carefully before giving their scores. The column explains what each hospital has done to make the programme effective; what it has failed to do, thus making the programme more ineffective; and what can be improved. Furthermore, the respondents could use Appendix II, which lists common workplace hazards in hospitals, as an additional guide indicating the hazards or health risk factors commonly found in each hospital.

To complete this instrument, circle the score 3, 2, 1, or 0 in the box provided to indicate the one that best matches the statement in the action item. You will have four choices:

• **Strongly agree -** indicates that the hospital routinely performs all the actions described and does so effectively (Three points given = 3).
• **Partially agree -** indicates that the hospital has performed all the actions as described, but only a few times effectively (Two points given = 2).
• **Strongly disagree -** indicates that the hospital has taken only some actions as described, and/or these have been ineffective or infrequent (One point given = 1).
• **No action -** indicates that the hospital has taken no action (No points given = 0).

The instrument allows Hospital OSH Units to calculate their own score and to summarise the scores for each section. The scores help users identify gaps and actions that, if taken, would improve the effectiveness of their hospital’s occupational health management system. It is recommended that hospitals complete the instrument periodically (e.g., annually or every six months) to track progress and improvement trends.

## Conclusions and suggestions

The implementation of occupational health service programmes in hospitals is an integral part of the efforts to protect workers and improve the quality of health services as a whole. Hospitals as workplaces have complex and high risk characteristics, both for health and non-health workers. Therefore, a systematic approach is needed to ensure that all work risks can be identified, assessed and controlled effectively.

This study aimed to develop an instrument that can be used to assess and evaluate the implementation of occupational health service programmes in hospitals, especially type B general hospitals. The instrument development process was conducted through a mixed approach, starting with the identification of risk factors based on the literature review, followed by in-depth interviews and field observations. The resulting instrument was then tested for validity and reliability through an FGD involving various stakeholders, to ensure that the tool was suitable for implementation in hospitals.

The findings show that all of the hospitals already have occupational health programmes, but that their implementation has not been optimal. It was found that there remain gaps in aspects of hazard identification; implementation of risk assessments; technical and administrative controls; and the provision of comprehensive occupational health services, including health promotion, specific protection, early detection and prompt treatment, disability limitation, and rehabilitation in terms of return to work programmes. In addition, worker participation and commitment from hospital leaders and management also play an important role in determining the success of programmes.

The instrument developed covers seven main dimensions: management support, worker participation, risk identification and assessment, hazard control, disease prevention, education and training, and programme evaluation. It is designed to be used independently by hospitals to assess the status of their occupational health programme implementation and to design necessary improvement steps.

In conclusion, the instrument is a strategic tool that can be used by hospitals to improve the effectiveness of occupational health programme implementation in a sustainable manner.

Routine application of the instrument is expected to provide an objective picture of implementation conditions in the field; to support data-based decision-making processes; and to become the basis for policy formulation at the institutional and national levels. Regulatory support, management commitment, and involvement of all hospital elements are key to realising a healthy, safe and productive workplace for the whole hospital community.

Last but not least, a unified national dashboard (with collaboration between the Ministry of Health, Ministry of Manpower, and BPJS Ketenagakerjaan) should be developed to monitor workplace accidents, occupational diseases, and near-misses in real-time, supported by pilot projects and digitalization, which can be addressed by adapting the OSHA/EU-OSHA model to the Indonesian context.

## Ethical consideration

This study has gone through a review process and was approved by the Institutional Review Board from teaching hospital Rumah Sakit Universitas Indonesia (approval number: S-091/KETLIT/RSUI/VII/2024). Written informed consent was obtained from all participants, including informants for in-depth interviews and stakeholders in focus group discussions (FGD). The consent process ensured that participants were fully informed about the study’s purpose, procedures, potential risks, and their right to withdraw at any time without consequences. No animal or human biological materials were involved in this study. All data were anonymized, and identifiable information was securely stored to protect participant confidentiality.

## Data Availability

The prototype instrument developed in this study, designed for assessing occupational health in hospitals, is publicly available on the Figshare data repository. The instrument is provided under a CC-BY 4.0 license, which permits its reuse and adaptation for future research, provided proper attribution is given. The instrument can be accessed at the following DOI: 10.6084/m9.figshare.30044053. The qualitative datasets supporting the findings of this study, including observational notes, in-depth interview transcripts, and focus group discussion records, are not publicly available to protect participant confidentiality and privacy. However, anonymized or summarized data may be made available upon reasonable request by contacting the corresponding author via email (Indri Hapsari Susilowati, indri@ui.ac.id). Requests will be evaluated to ensure compliance with ethical guidelines and participant consent agreements. Supplementary materials, such as interview guides and discussion protocols, can also be provided where appropriate. Researchers seeking access must outline their intended use of the data to facilitate review. Appendix data can be found in https://doi.org/10.6084/m9.figshare.30044053 Data are available under the terms of the Creative Commons Attribution 4.0 International license (CC-BY 4.0).
