# Supplementary material for: Development of an Instrument for Evaluating the Implementation of Occupational Health Programmes in Hospitals
Source: F1000Res. 2025 Sep 29;14:1009. [Version 1] doi: 10.12688/f1000research.165842.1 (PMC12699208; doi:10.12688/f1000research.165842.1)
Supplement: Peer review history

# Reviewer report: Reviewer response for version 1

**DOI:** 10.5256/f1000research.182587.r432381

**Published:** 2025-12-26

**Authors:** Ioannis Pantelis Adamopoulos

**Affiliations:** 1 University of West Attica, Athens, Greece

Congratulations to the authors; this is a significant study that highlights an important issue regarding occupational risks in hospitals and the hazards faced by all healthcare workers.

 Few works globally offer tools to minimize and prevent these hazards.

 While this study is valuable, it is deemed appropriate as a literature review rather than a research study, providing the development of an instrument for evaluating the implementation of occupational health programs in hospitals. My decision is to accept the manuscript (as review study) as it is, along with my comments.

Is the work clearly and accurately presented and does it cite the current literature?

Yes

If applicable, is the statistical analysis and its interpretation appropriate?

Yes

Are all the source data underlying the results available to ensure full reproducibility?

Yes

Is the study design appropriate and is the work technically sound?

Yes

Are the conclusions drawn adequately supported by the results?

Yes

Are sufficient details of methods and analysis provided to allow replication by others?

Yes

Reviewer Expertise:

Yes, it is innovative!

I confirm that I have read this submission and believe that I have an appropriate level of expertise to confirm that it is of an acceptable scientific standard.

# Reviewer report: Reviewer response for version 1

**DOI:** 10.5256/f1000research.182587.r432386

**Published:** 2025-12-11

**Authors:** Cheryl Lin Rielander

**Affiliations:** 1 University of South Africa, Pretoria, Gauteng, South Africa

**EVALUATION REPORT**

*Article: Development of an Instrument for Evaluating the Implementation of Occupational Health Programmes in Hospitals*

 Review Date: November 28, 2025

 Thank you for the opportunity to review this article, which addresses a critical aspect of occupational health in hospital settings. This review offers an opportunity to evaluate the clarity, methodological rigor, and practical applicability of the proposed instrument, as well as its potential to contribute to improved health outcomes and informed policy development.

**Executive Summary**

 This report evaluates the article titled 'Development of an Instrument for Evaluating the Implementation of Occupational Health Programmes in Hospitals'. The study aimed to develop a tool for assessing the implementation of occupational health services in hospitals in Indonesia. Using a qualitative exploratory design, the authors conducted literature reviews, interviews, observations, and focus group discussions to generate and validate the instrument. The instrument consists of seven sections and 53 statements, covering management support, worker participation, hazard identification, health hazard control, disease prevention, education and training, and programme evaluation. Although the recommendations provided are advantageous, they do not provide sufficient detail on the practical steps for implementing these recommendations in hospitals. In addition, the validation process for the developed instrument was limited in scope, relying primarily on a single focus group discussion rather than broader, multi-site validation.

 For future research or related systematic literature reviews SLRs, the authors may consider using the PRISMA (Preferred Reporting Items for SLRs and Meta-Analyses) framework, as it provides a standardised methodology for conducting and reporting on SLRs. This ensures transparency, reproducibility, and comprehensive coverage of relevant studies. Incorporating PRISMA would strengthen the rigor of the SLR component, particularly if the study aims to synthesize evidence on occupational health programme effectiveness or similar interventions.”

 The study concludes that the instrument can serve as a strategic tool for improving occupational health programmes in hospitals. However, its effectiveness and applicability across different hospital settings require further testing through pilot implementation and multi-site validation to ensure reliability, adaptability, and scalability.

**Detailed Evaluation**

**Limitations**

**Limited Sample Size and Scope**

 The study was conducted only in three Type B hospitals in Indonesia, excluding Type A and Type C hospitals, which may have different organizational structures, resources, and challenges. This limits the generalisability of the findings.

**Lack of Quantitative Data**

 The research relies primarily on qualitative methods (literature review, interviews, observations, and focus group discussions. The absence of quantitative data reduces the ability to statistically validate findings and limits the robustness of generalisations.

**Validation Process Constraints**

 The instrument was validated through a single 2-day Focus Group Discussion with selected stakeholders. Broader validation across multiple hospitals and regions would enhance reliability and acceptance.

**Instrument Testing Limitations**

 While the instrument is proposed as a strategic tool, its effectiveness and adaptability were not tested through a pilot or longitudinal study. As such, further testing is recommended to confirm usability, scalability, credibility, and reliability.

- **Usable** (practical and easy to apply in real settings),
- **Scalable** (adaptable across different hospital types and resource levels),
- **Credible** (accepted by stakeholders and aligned with best practices),
- **Reliable** (produces consistent results across applications).

**Exclusion of Diverse Resource Settings**

 Practical steps for implementing recommendations in hospitals with varying resource conditions were not detailed, which may affect their applicability in Type A and Type C hospitals, as well as in low-resource settings.

**Regulatory Context Changes**

 The study references Government Regulation No. 88 of 2019, which provides detailed provisions for implementing occupational health in the workplace, covering areas such as disease prevention, health improvement, and health management. This regulation should be read in conjunction with Law No. 11 of 2020, as amended by Law No. 6 of 2023, in relation to the Job Creation Law, which streamlines regulations and enhances worker protection and welfare. 

**1. Is the work clearly and accurately presented, and does it cite the current literature?**

 Yes. The article is well-structured and cites relevant and current literature, including WHO/ILO global estimates and Indonesian regulations. References include recent studies on occupational health and COVID-19 impacts on healthcare workers.

 However, an expansion on the Government Regulation No. 88 of 2019 and its link with Law No. 11 of 2020, as amended by Law No. 6 of 2023, could add more value.

**2. Is the study design appropriate, and is the work technically sound?**

 Yes, partially. The qualitative exploratory design is appropriate for developing an assessment instrument. The use of thematic analysis and adaptation of OSHA’s questionnaire ensures technical soundness.

 However, The absence of quantitative data reduces the ability to statistically validate findings and limits the robustness of generalisations.

**3. Are sufficient details of methods and analysis provided to allow replication by others?**

 Yes. The article provides detailed steps: literature review, interviews, observations, thematic analysis, and FGD validation. Appendices include the instrument and guidelines, enabling replication.

**4. If applicable, is the statistical analysis and its interpretation appropriate?**

 Not applicable. The study is qualitative and does not involve statistical analysis. Semi-quantitative scoring is explained clearly for the instrument.

 The absence of quantitative data reduces the ability to statistically validate findings and limits the robustness of generalisations.

**5. Are all the source data underlying the results available to ensure full reproducibility?**

 Partially. The instrument is publicly available on Figshare. Qualitative data (interview transcripts, focus group discussion notes) are not fully open but can be requested under ethical guidelines.

**6. Are the conclusions drawn adequately supported by the results?**

 Yes. The conclusions presented in the study are well-aligned with the findings. While hospitals have established occupational health programmes, the evidence indicates significant gaps in hazard control measures, comprehensive risk assessments, and leadership commitment to programme implementation. These shortcomings highlight the need for stronger governance structures and proactive management involvement. The recommendations for routine use of the developed instrument are particularly relevant, as they provide a systematic approach for monitoring and improving programme effectiveness over time. Furthermore, the suggestion to integrate the instrument into policy development processes addresses the identified gaps by promoting standardization, accountability, and continuous improvement across healthcare facilities. Recommendations for routine instrument use and policy development are consistent with identified gaps. By linking these recommendations to the observed deficiencies, the study demonstrates a logical progression from problem identification to actionable solutions, reinforcing its practical and strategic value.

 Clearer indication of future studies should prioritise multi-site validation of the instrument across diverse hospital types (A, B, and C) to ensure generalisability. Furthermore, integrating quantitative methods with qualitative approaches will strengthen reliability and enable the statistical validation of findings.

**Strengths, Weaknesses, and Recommendations**

**Strengths:**

 - Clear presentation and logical structure

 - Comprehensive literature review

 - Detailed methodology enabling replication

 - Practical and relevant recommendations

**Weaknesses:**

 - Limited sample size (three hospitals)

 - Lack of quantitative data

 - Validation limited to focus group discussions

 - No conceptual framework linking findings to theory

**Recommendations:**

 - Expand sample size and include Type A and C hospitals

 - Incorporate quantitative data for stronger generalization - Broaden validation with more stakeholders

 - Develop a conceptual framework linking findings to CSR and occupational health theories

**Suggestions for Future Research**

 - Conduct mixed-method studies combining qualitative and quantitative approaches

 - Validate the instrument across diverse hospital types

 - Explore the impact of the instrument on policy and practice

 - Develop digital dashboards for real-time monitoring of occupational health indicators

 Thank you

Is the work clearly and accurately presented and does it cite the current literature?

Yes

If applicable, is the statistical analysis and its interpretation appropriate?

No

Are all the source data underlying the results available to ensure full reproducibility?

Partly

Is the study design appropriate and is the work technically sound?

Partly

Are the conclusions drawn adequately supported by the results?

Yes

Are sufficient details of methods and analysis provided to allow replication by others?

Yes

Reviewer Expertise:

Occupational health, occupational health and safety, occupational risk exposure, risk and strategic safety management

I confirm that I have read this submission and believe that I have an appropriate level of expertise to confirm that it is of an acceptable scientific standard.

# Reviewer report: Reviewer response for version 1

**DOI:** 10.5256/f1000research.182587.r423104

**Published:** 2025-11-04

**Authors:** Anita Camellia, Tan Malaka

**Affiliations:** 1 Universitas Sriwijaya, Sumatra, Indonesia; 2 Ilmu Kesehatan Masyarakat dan Ilmu Kedokteran Komunitas, Universitas Sriwijaya Fakultas Kedokteran, Palembang, South Sumatra, Indonesia

1. The article presents a significant effort in developing an instrument to evaluate the implementation of occupational health programs in hospitals. ​ However, several limitations can be identified that may impact the comprehensiveness and applicability of the findings. Firstly, the study lacks in-depth quantitative data, which could strengthen the analysis and provide a more robust basis for generalization. Additionally, the sample size is limited to three Type B hospitals in Indonesia, which may not adequately represent the broader context of occupational health program implementation across all hospitals in the country.  While the recommendations provided are valuable, the article does not sufficiently detail the practical steps for implementing these recommendations in hospitals with varying resource conditions. Furthermore, the validation of the developed instrument was conducted through a focus group discussion (FGD), but broader validation involving more hospitals and stakeholders would enhance its reliability and acceptance. ​

 The focus on Type B hospitals excludes Type A and Type C hospitals, which may have different characteristics and challenges, thereby limiting the scope of the study. ​ Lastly, the article is awaiting peer review, meaning its scientific validity and quality have not yet been fully verified by experts in the field. ​

 These limitations highlight areas for improvement in future research, including expanding the sample size, incorporating quantitative data, providing practical implementation strategies, conducting broader validation, and assessing the impact of the instrument. Addressing these gaps would enhance the comprehensiveness, reliability, and applicability of the findings. 

 2. Government Regulation No.88 of 2019 (Page 3) is no longer in use and has been replaced by another regulation.

 3. Page 4. We have to consider that using absolute numbers of cases is inappropriate to conclude the rise of accident frequency since the number of workers and company was also on the rise from year to year.

Is the work clearly and accurately presented and does it cite the current literature?

Yes

If applicable, is the statistical analysis and its interpretation appropriate?

Not applicable

Are all the source data underlying the results available to ensure full reproducibility?

Yes

Is the study design appropriate and is the work technically sound?

Yes

Are the conclusions drawn adequately supported by the results?

Yes

Are sufficient details of methods and analysis provided to allow replication by others?

Yes

Reviewer Expertise:

Occupational Safety and Health

We confirm that we have read this submission and believe that we have an appropriate level of expertise to confirm that it is of an acceptable scientific standard.
